# Editorial

**Published:** 2014-06-12

**Authors:** Shibal Bhartiya, Tarek Shaarawy, Tanuj Dada

‘But medicine has long had all its means to hand, and has discovered both a principle and a method, through which the discoveries made during a long period are many and excellent, while full discovery will be made, if the inquirer be competent, conduct his researches with knowledge of the discoveries already made, and make them his starting-point. But anyone who, casting aside and rejecting all these means, attempts to conduct research in any other way or after another fashion, and asserts that he has found out anything, is and has been victim of deception.’

—Hippocrates

One of the commonest problems of glaucoma filtration surgery is bleb scarring. The advent of antifibrotic agents, vascular endothelial growth factor (VEGF) inhibition as well as newer pharmacological compounds and materials has improved the predictability and longevity of filtration surgeries. Gaskin et al discuss and critically evaluate the application of antifibrotic agents in glaucoma filtration surgery in a comprehensive two-part review.

Continuing the theme of subconjunctival scarring being an important determinant of glaucoma surgery outcomes, Howlett et al assessed bulbar conjunctival and Tenon’s layer thickness using optical coherence tomography (OCT) in a pilot study designed to eventually help predict surgical success.

Jung et al compare the efficacy of different surgical strategies for intraocular pressure (IOP) control in Hispanic glaucoma patients with and without visually significant cataracts and conclude that trabeculectomy and phacotrabeculectomy are both viable surgical options for managing open angle glaucoma with similar rates of success, IOP reduction, decrease in use of IOP-lowering medications and postoperative complication rates.

Given that patients with glaucoma often lose vision despite adequate medical or surgical treatment, complementary and alternative medicine deserves scientific scrutiny from glaucoma surgeons. Bhartiya et al critically review the currently available evidence, most of which remains empirical and anecdotal in nature.

Prado et al investigate the treatment outcomes of argon laser peripheral iridoplasty (ALPI) in angle closure mechanisms other than pupillary block concluding that it is a useful procedure, independent of the underlying mechanism, leading to angle widening and moderate IOP reduction in most cases.

The demographical profile, presentation, management and outcome of traumatic glaucoma in children as well as the various factors associated with advanced glaucomatous changes are elucidated in a comprehensive review by Kaur et al.

Agarwal et al evaluate the normative data of macular thickness and retinal nerve fiber layer thickness among normal subjects using spectral domain optical coherence tomography in a central Indian population, reiterating that values obtained by time domain OCT are not comparable with those of the spectral domain OCT.

We hope you find this issue relevant to your day-to-day clinical practice and look forward to your feedback.

Best wishes

**Shibal Bhartiya**

**Tarek Shaarawy**

**Tanuj Dada**

